# LNKs-RVEs complex ticks in the circadian gating of plant temperature stress responses

**DOI:** 10.1007/s44154-023-00113-1

**Published:** 2023-08-16

**Authors:** Xiaodong Xu, Qiguang Xie

**Affiliations:** State Key Laboratory of Crop Stress Adaptation and Improvement, School of Life Sciences, Henan University, Kaifeng, 475004 China

**Keywords:** Circadian clock, LNKs, RVEs, Heat stress, Cold resistance

## Abstract

Recently, Kidokoro et al. found that protein complex LNK3,4-RVE4,8 and LNK1,2-RVE4,8 of the circadian clock modulates plant cold- and high-temperature tolerance, respectively. Here, we reviewed the discovery of LNKs, the dynamically formed morning-phased clock complexes, and their critical role on endogenous circadian rhythms. In addition, we summarized the research work on LNKs with the interacting proteins RVEs, CCA1 in temperature responses and discussed how the circadian clock confer increased fitness via gating the rhythmic expression of their target genes.

The circadian clock is a cell-autonomous timekeeping mechanism that readily transduces cyclic changes in external cues (time giver or *Zeitgebers*) into rhythmic physiological and biochemical processes, thereby achieving the adaptation to photoperiod and ambient temperatures of living organisms. In the model plant *Arabidopsis thaliana*, Myb-like transcription factors CIRCADIAN CLOCK-ASSOCIATED 1 (CCA1), LATE ELONGATED HYPOCOTYL (LHY), REVEILLEs (RVEs), and PSEUDO-RESPONSE REGULATORs (PRR9, PRR7) transcripts and proteins are enriched in the daytime, while PRR5, PRR3, PRR1/TOC1 (TIMING OF CAB EXPRESSION1), ELF3-ELF4-LUX (known as Evening Complex, EC) reach their expression peaks in the evening or at night. These core oscillators expressed during the 24-h day constitute the multiple interlocked transcriptional-translational feedback loops (TTFLs) of the circadian clock. The 6% of more than 8,000 Arabidopsis genes on high-density oligonucleotide microarrays show rhythmic enrichment, and up to 89% of Arabidopsis transcriptome is modulated by the circadian clocks, light and temperature cycles, and this fact has been confirmed in the high-throughput transcriptome sequencing of crops including corn, rice and soybean (Harmer et al. 2000; Michael et al. 2008; Filichkin et al. 2011; Li et al. 2020). Thus, the circadian core oscillators gating or spatiotemporal fine-tuning regulates the vast majority of time-dependent gene expression, protein activity, physiological processes, and agronomic traits.

Two members of the NIGHT LIGHT–INDUCIBLE AND CLOCK-REGULATED family, LNK1 and LNK2, were originally identified as being light-induced at midnight, and both are involved in regulating the pace of red light intensity-dependent circadian rhythm, as well as hypocotyl elongation and flowering time (Rugnone et al. 2013; Xie et al. 2014). *LNK1* and *LNK2* promoter activity (*LNK1:LUC* and *LNK2:LUC*) and the enrichment of their proteins (*LNK1:LNK1-LUC* and *LNK2:LNK2-LUC*) showed circadian rhythmicity with peaks occurring in the morning (Xie et al. 2014). LNK1 and LNK2 dynamically interacted with CCA1, LHY, RVE4, and RVE8, respectively, during the daytime. The morning complex LNK1,2-RVE4,8 activates the transcription of *PRR5* and *TOC1*, of which LNK1 and LNK2 act as coactivators (Xie et al. 2014). In addition, LNK1 and LNK2 also interact with RNA Polymerase II and the transcript elongation FACT complex to determine the RNA Pol II and H3K4me3 deposition at the *PRR5* and *TOC1* loci and target mRNA circadian oscillations (Ma et al. 2018). The LNK family has four members that share conserved protein sequences, of which LNK1 and LNK2 have a molecular weight of about 66 kDa, while LNK3 and LNK4 look like truncated proteins with a molecular weight of about 30 kDa. Unlike *lnk1* and *lnk2*, the parameters of circadian rhythm in *lnk3-1* and *lnk4-1* were not altered under free-running conditions (constant light, 22 °C) (Xie et al. 2014). However, the *lnk1 2 3 4* quadruple mutant (*lnkQ*) displayed a 1.3-h lengthened period of circadian rhythm than *lnk1 lnk2* double mutant, and it was more significant in rosette size and biomass than the *lnk1 lnk2* plant. (de Leone et al. 2019). Therefore, the function of *LNK3* and *LNK4* in the circadian clock system was still largely unknown.

Recently, Kidokoro et al. found that LNK3 and LNK4 activate the expression of the clock gene *PRR5* at lower temperature (4 °C), and that the LNK3,4-RVE4,8 complex and the LNK1,2-RVE4,8 complex determine plant temperature stress tolerance by regulating the expression of cold- or heat-responsive genes, respectively (Kidokoro et al. 2023). In this study, *PRR5* transcript level at 22 °C was significantly repressed in *lnk1 lnk2* double mutant and was consistent in *lnk3 lnk4* and wild-type (Col-0) plant, indicating that LNK1 and LNK2 as an activator is regulating *PRR5* transcription as previously known. At chilling conditions (4 °C), LNK1 and LNK2 completely lost their regulation of *PRR5* expression, as *PRR5* transcript levels are the same as in the wild type. Of interest, cold stress contributes to the enrichment of *PRR5* transcripts in *lnk3 lnk4* by about 40% of that in wild type, suggesting that LNK3 and LNK4 show an activating effect on *PRR5* expression in response to temperature changes. This finding reveals a temperature-dependent function of LNK3 and LNK4 in the circadian clock LNKs-PRR5 loop. Next, the circadian rhythms need to be measured to know whether LNK3, LNK4 and PRR5 determine the clock pace, amplitude, or phase in cold conditions.

This article spotlights the selective interaction of LNK1, LNK2 or LNK3, LNK4 with the circadian oscillator RVE4,8 and the response of LNKs-RVEs protein complexes to environmental cold or heat stress. The results showed that all four LNKs could be induced by low temperature signals (4 °C), while the *LNK3* and *LNK4* transcript levels were significantly higher than their expression at 22 °C, which was different from that of *LNK1* and *LNK2*. The DREB1A/CBF3, DREB1B/CBF1 and DREB1C/CBF2 in Arabidopsis are known to be transcription factors rapidly induced by cold and freezing treatment, and they enhance low temperature stress tolerance by activating the expression of cold response (*COR*) genes with promoter-containing CRT/DRE cis-elements (Jaglo-Ottosen et al. 1998). Kidokoro et al. found that the cold-induced *DREB1A* expression was similar in wild-type and *lnk1 lnk2* plants, whereas it was reduced by more than 50% in *lnk3 lnk4* and *lnk1 lnk2 lnk3 lnk4/lnkQua* mutants. Previously, Kidokoro et al. revealed RVE4 and RVE8 protein accumulation in the nucleus in response to chilling/4 °C and that RVE4 and RVE8 protein binds more to *DREB1A* promoter to activate its expression under cold stress condition than CCA1 and LHY (Kidokoro et al. 2021). In the current article, the expression of cold-induced *DREB1A* was also reduced by 60–80% in *rve4 rve8* mutant (Kidokoro et al. 2023). After deletion analysis of the C- and N-terminal regions of RVE8 protein, it was found that the C-terminal of RVE8 determined its regulation of *DREB1A* expression under cold stress. This study also confirmed protein interactions between LNK3,4 and RVE4,8 in the nuclei at both 24 °C and 4 °C conditions. Importantly, transcriptome sequencing of *lnkQua* and wild-type plants treated with 4 °C for 3 h was performed to help identify the LNKs-regulated signaling pathways and hundreds of target genes including *COR15A* and *COR47*. Further physiological phenotypic analysis of -10 °C treatment in 4 °C/cold-acclimated plants confirmed RVEs and LNKs as regulating cold-induced freezing tolerance. Another highlight of this study is that the authors found phosphorylation of LNK3 and LNK4 proteins and an increased amount of phosphorylated LNK3 and LNK4 after 3-h 4 °C treatment. In summary, this article identified the LNK3,4-RVEs module, which plays a critical role in *COR* gene expression and low-temperature tolerance of plants.

On the other hand, in this study, heat stress-induced *ETHYLENE RESPONSIVE FACTORS 53*/*ERF53* and *ERF54* gene expression was more repressed in *lnk1 lnk2* than in *lnk3 lnk4* plants in response to heat stress (37 °C), suggesting the major contribution of LNK1 and LNK2 in high temperature tolerance. Further in the analysis of 45-min 43 °C heat shock after 37 °C acclimation, *lnk1 lnk2* plants displayed more tolerance than *lnk3 lnk4* mutant. This result provides the working hypothesis that the LNK1,2-RVE4,8 complex may dominate the heat shock response (HSR), distinct from the role of LNK3,4-RVE4,8 in cold and freezing resistance (Fig. 1).

**Fig. 1:**
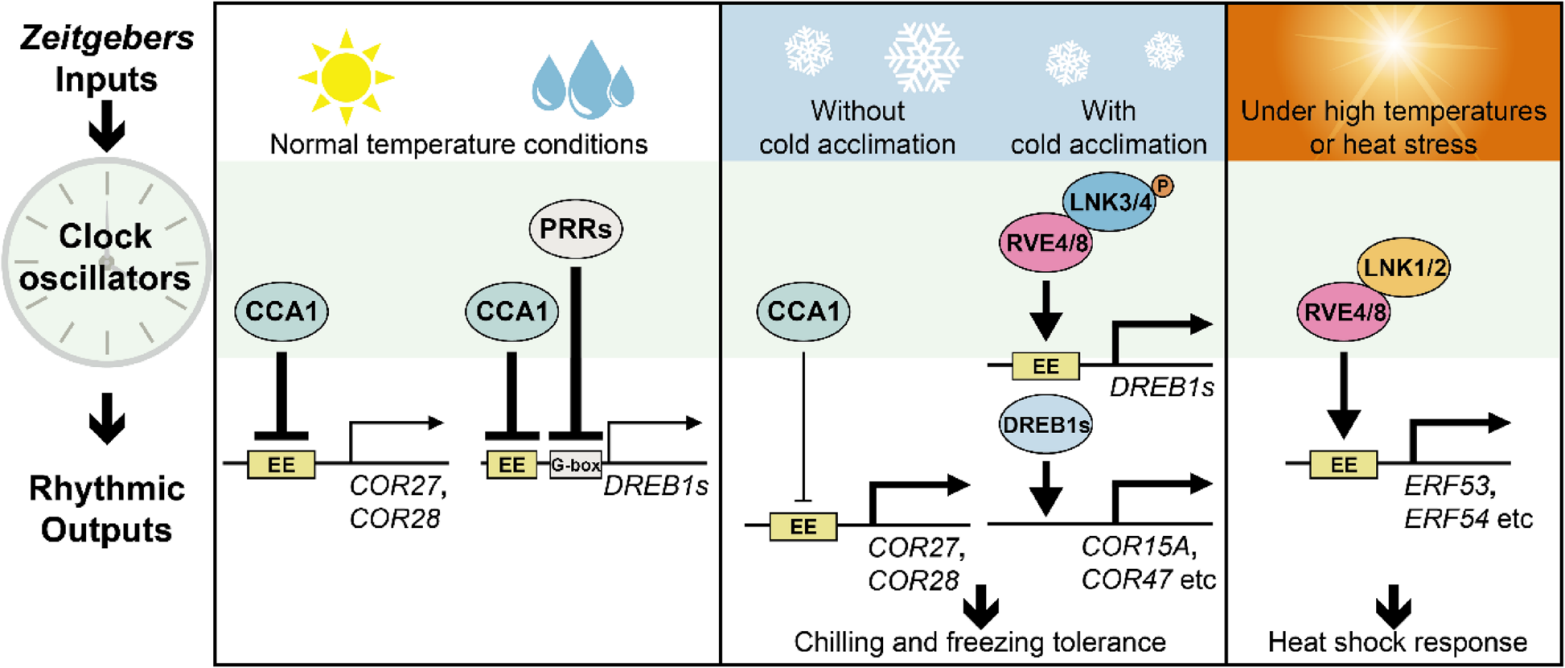
Circadian oscillators respond to the fluctuations in ambient temperature. Circadian transcription factors RVE4, RVE8 and coactivators LNK3, LNK4 activate *DREB1s*, *COR15A*, and *COR47* expression to achieve the chilling and freezing tolerance. Cold-induced phosphorylation of LNK3,4 proteins contribute to the low temperature response of the RVE4,8-LNK3,4 complex. In response to heat shock and thermal stress, RVE4 and RVE8 recruit LNK1 and LNK2 and activate *ERFs* expression to obtain thermotolerance. In addition, the LNK1,2-interacting protein CCA1 directly regulate the transcription of *COR27* and *COR28*

The protein–protein interactions between the circadian core oscillators show a 24-h rhythmicity, predicting that the temporal window of peak expression of the morning-phased or evening-phased protein complex are associated with the anticipation of fluctuations in environmental signals. This article revealed that protein complexes composed of LNK1,2 and LNK3,4 recruit RVEs with distinct sensitivity to temperature fluctuations; this finding contributes to the understanding of the molecular and physiological functions of transcriptional cofactors, including LNKs. It is also worth exploring the role of LNK1-4 in the transcriptional regulation of their target genes, as LNKs interact with transcriptional activators RVEs along with transcriptional repressors CCA1 and LHY, and these protein complexes are all enriched in the early morning. It is known that dysfunction of *PRR9*, *PRR7*, and *COR27*, *COR28* enhances freezing tolerance (-7 °C) without cold assimilation; and PRR9 and PRR7 suppress *CCA1* expression in TTFLs of the circadian clock (Wang et al. 2017). Cold stress attenuates CCA1 binding to the *COR27* and *COR28* promoter, which potentially be involved in the low temperature response in a CBF-independent pathway. Recently, Sorkin et al. identified the RVE8-LNK1-COR27,28 protein complex by time-of-day affinity purification-mass spectrometry, and found that LNK1 and LNK2 expression respond to temperature entrainment (Sorkin et al. 2023).

Circadian clock components provide the genetic basis for crops adaptation to agricultural environments. For example, *LNK2* was found to co-localize with the circadian period length QTL in cultivated tomato (Muller et al. 2018). The frequency of *LNK2* deletion increased during tomato domestication, which slowed down the clock pace, i.e., partial mutation of *LNK2* lengthened the period length of endogenous circadian rhythms. Muller et al. also revealed the evolutionary trajectory of *EMPFINDLICHER IM DUNNKELROTEN LICHT 1* (*EID1*) and *LNK2* mutations during domestication and breeding with the analysis of a subset of 274 tomato accessions. *EID1* encodes an F-box protein that acts in the PHYA-dependent light signaling pathway in Arabidopsis. Soybean GmEID1 interacts with J (a homolog of ELF3), which regulates photoperiodic flowering and yield in a variety of crops, including soybean, barley, and rice (Faure et al. 2012; Andrade et al. 2022; Qin et al. 2023). In summary, the mechanism of circadian oscillator protein complex in the response to environmental timing cues (inputs) and clock-gating physiological processes (outputs) deserves further investigation.

## Data Availability

Not applicable.

## Abbreviations

CBF1: C-repeat/DRE binding factor 1
CCA1: CIRCADIAN CLOCK ASSOCIATED 1
COR: Cold-responsive gene
DREB1A: DEHYDRATION RESPONSE ELEMENT B1A
EC: Evening complex; EID1 (EMPFINDLICHER IM DUNNKELROTEN LICHT 1)
ELF3: EARLY FLOWERING 3
ERF: ETHYLENE RESPONSIVE FACTORS
HSR: HEAT SHOCK RESPONSE
LHY: LATE ELONGATED HYPOCOTYL
LNK1: NIGHT LIGHT-INDUCIBLE AND CLOCKREGULATED 1
LUX: LUX ARRHYTHMO
PRRs: PSEUDO-RESPONSE REGULATORs
RVE4: REVEILLE 4
TOC1: TIMING OF CAB EXPRESSION 1
TTFLs: Transcriptional-translational feedback loops
ZTL: ZEITLUPE

## References

[CR1] Andrade L, Lu Y, Cordeiro A, Costa JMF, Wigge PA, Saibo NJM et al (2022) The evening complex integrates photoperiod signals to control flowering in rice. Proc Natl Acad Sci USA. 119:e2122582119. 10.1073/pnas.2122582119 10.1073/pnas.2122582119 PMC9245669 35733265

[CR2] de Leone MJ, Hernando CE, Romanowski A, Garcia-Hourquet M, Careno D, Casal J (2019). The LNK gene family: at the crossroad between light signaling and the circadian clock. Genes (basel).

[CR3] Faure S, Turner AS, Gruszka D, Christodoulou V, Davis SJ, von Korff M (2012). Mutation at the circadian clock gene EARLY MATURITY 8 adapts domesticated barley (Hordeum vulgare) to short growing seasons. Proc Natl Acad Sci USA.

[CR4] Filichkin SA, Breton G, Priest HD, Dharmawardhana P, Jaiswal P, Fox SE et al (2011) Global profiling of rice and poplar transcriptomes highlights key conserved circadian-controlled pathways and cis-regulatory modules. PLoS ONE 6:e16907. 10.1371/journal.pone.0016907 10.1371/journal.pone.0016907 PMC3111414 21694767

[CR5] Harmer SL, Hogenesch JB, Straume M, Chang HS, Han B, Zhu T (2000). Orchestrated transcription of key pathways in Arabidopsis by the circadian clock. Science.

[CR6] Jaglo-Ottosen KR, Gilmour SJ, Zarka DG, Schabenberger O, Thomashow MF (1998). Arabidopsis CBF1 overexpression induces *COR* genes and enhances freezing tolerance. Science.

[CR7] Kidokoro S, Hayashi K, Haraguchi H, Ishikawa T, Soma F, Konoura I et al (2021) Posttranslational regulation of multiple clock-related transcription factors triggers cold-inducible gene expression in Arabidopsis. Proc Natl Acad Sci USA 118:e2021048118. 10.1073/pnas.2021048118 10.1073/pnas.2021048118 PMC7958436 33649234

[CR8] Kidokoro S, Konoura I, Soma F, Suzuki T, Miyakawa T, Tanokura M, Shinozaki K, Yamaguchi-Shinozaki K (2023) Clock-regulated coactivators selectively control gene expression in response to different temperature stress conditions in Arabidopsis. Proc Natl Acad Sci USA 120:e2216183120. 10.1073/pnas.2216183120 10.1073/pnas.2216183120 PMC10120023 37036986

[CR9] Li C, Li YH, Li Y, Lu H, Hong H, Tian Y (2020). A Domestication-associated gene GmPRR3b regulates the circadian clock and flowering time in soybean. Mol Plant.

[CR10] Ma Y, Gil S, Grasser KD, Mas P (2018). Targeted recruitment of the basal transcriptional machinery by LNK clock components controls the circadian rhythms of nascent RNAs in Arabidopsis. Plant Cell.

[CR11] Michael TP, Mockler TC, Breton G, McEntee C, Byer A, Trout JD et al (2008) Network discovery pipeline elucidates conserved time-of-day-specific cis-regulatory modules. PLoS Genet 4:e14. 10.1371/journal.pgen.0040014 10.1371/journal.pgen.0040014 PMC2222925 18248097

[CR12] Muller NA, Zhang L, Koornneef M, Jimenez-Gomez JM (2018). Mutations in EID1 and LNK2 caused light-conditional clock deceleration during tomato domestication. Proc Natl Acad Sci USA.

[CR13] Qin C, Li H, Zhang S, Lin X, Jia Z, Zhao F et al (2023) GmEID1 modulates light signaling through the Evening Complex to control flowering time and yield in soybean. Proc Natl Acad Sci USA 120:e2212468120. 10.1073/pnas.2212468120 10.1073/pnas.2212468120 PMC10104576 37011215

[CR14] Rugnone ML, Faigón Soverna A, Sanchez SE, Schlaen RG, Hernando CE, Seymour DK (2013). LNK genes integrate light and clock signaling networks at the core of the Arabidopsis oscillator. Proc Natl Acad Sci USA.

[CR15] Sorkin ML, Tzeng SC, King S, Romanowski A, Kahle N, Bindbeutel R (2023). COLD REGULATED GENE 27 and 28 antagonize the transcriptional activity of the RVE8/LNK1/LNK2 circadian complex. Plant Physiol.

[CR16] Wang P, Cui X, Zhao C, Shi L, Zhang G, Sun F (2017). COR27 and COR28 encode nighttime repressors integrating Arabidopsis circadian clock and cold response. J Integr Plant Biol.

[CR17] Xie Q, Wang P, Liu X, Yuan L, Wang L, Zhang C (2014). LNK1 and LNK2 are transcriptional coactivators in the Arabidopsis circadian oscillator. Plant Cell..

